# α-Aminophosphonate Derivatives for Enhanced Flame Retardant Properties in Epoxy Resin

**DOI:** 10.3390/ma14123230

**Published:** 2021-06-11

**Authors:** Melissa K. Stanfield, Jeronimo Carrascal, Luke C. Henderson, Daniel J. Eyckens

**Affiliations:** 1 Carbon Nexus, Institute for Frontier Materials, Deakin University, Waurn Ponds, Geelong, VIC 3216, Australia; luke.henderson@deakin.edu.au; 2 School of Civil Engineering, University of Queensland, St. Lucia, QLD 4072, Australia; j.carrascaltirado@uq.edu.au; 3 CSIRO Manufacturing, Clayton, VIC 3168, Australia

**Keywords:** flame retardant, epoxy resin, phosphorus, fluorine, flammability, limiting oxygen index

## Abstract

This work demonstrates the introduction of various α-aminophosphonate compounds to an epoxy resin system, thereby improving flame retardance properties. The α-aminophosphonate scaffold allows for covalent incorporation (via the secondary amine) of the compounds into the polymer network. This work explores the synergistic effect of phosphorus and halogens (such as fluorine) to improve flame retardancy. The compounds were all prepared and isolated in analytical purity and in good yield (95%). Epoxy samples were prepared, individually incorporating each compound. Thermogravimetric analysis showed an increased char yield, indicating an improved thermal resistance (with respect to the control sample). Limiting oxygen index for the control polymer was 28.0% ± 0.31% and it increased to 34.6% ± 0.33% for the fluorinated derivative.

## 1. Introduction

The increasing demand of structural resins for potential use in composites across industrial applications has allowed epoxy resins to find use in areas such as coatings, adhesives, and laminates. They possess advantageous properties such as mechanical strength, electrical insulating, and high chemical resistance allowing them to suit a substantial number of applications. However, their high flammability and low thermal stability are limitations that prevent their use in further applications. The flammability of epoxy resins is a major limitation of these materials, and improving this property is vital for their continued future use in industry [1,2,3].

Phosphorus-based organic compounds are known to be highly effective flame retardants [4,5,6,7,8]. Phosphorus has the ability to promote formation of a char layer and/or a flame inhibitor. Wang et al. reported the improved flame retardance of epoxy resin system (bisphenol A diglycidyl ether (DGEBA)/diamino diphenyl methane (DDM)), similar to that used in this work. In that work, the authors modified the resin system to introduce phosphorus, reporting an excellent flame retardancy with UL-94 V0 rating and Limiting Oxygen Index (LOI) of ~32.8% [9]. When Luo et al. introduced 5,10-dihydro-phenophosphazine-10-oxide (DPPA) to a DGEBA/DDM system as a co-curing agent at 2.5 wt %, a LOI of 33.6% was achieved [10].

There are reports in the literature of phosphorus-based flame retardants acting synergistically with elements such as sulphur, nitrogen, and silicon, to further improve the thermal resistance of polymeric materials [11,12,13,14,15].

The inclusion of halogenated species (Br, Cl, etc.) to flame retardant material is nowadays avoided due to the associated toxicity repercussions; however, fluorine has been recently reported to participate synergistically with phosphorus in flame retardant applications [11]. Fluorine-containing epoxy resins have found potential application as electronic packing material due to superior hydrophobic properties and low dielectric constant [16].

Flame retardants are introduced as additives [17,18,19,20] to the polymeric system or they can be covalently incorporated [21,22], by which they integrate into the polymer backbone. Covalent integration into the polymer network is useful to minimize deleterious effects on the mechanical properties of the material.

**Figure 1:**
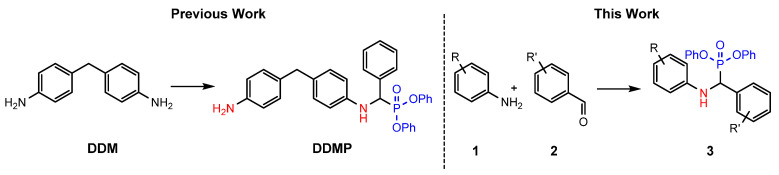
Scaffold of α-aminophosphonates employed in previous work and in this work; amine able to participate in cross-linking with the epoxy matrix is shown in red and the phosphonate moiety to introduce flame retardancy is shown in blue.

Our previous work reported the incorporation of phosphorus-containing compounds into an epoxy polymer network. The compounds were strategically designed to have a primary and secondary amine present, which allowed for the reaction and incorporation into the epoxy resin-based system (Figure 1). Concurrently introducing phosphorus atoms into the epoxy resin system. [23].

The work presented here utilizes the same easily accessible, one-pot synthesis, extending to incorporate a catalogue of analogues [24]. The effects of nitrogen, phosphorus, and halogenated compounds are used to explore their abilities to act synergistically, exploiting the efficiency of halogens but at extremely low concentrations.

Herein, we present a significant improvement in the self-extinguishment of epoxy resin with the incorporation of a catalogue of α-aminophosphonate compounds. An improvement in flame retardancy has been observed through thermogravimetric analysis (TGA), indicating a higher char yield for the modified samples and an increase in limiting oxygen index (LOI). Near infrared spectroscopy (NIR) data confirmed the successful covalent incorporation of the compounds into the resin system.

## 2. Materials and Methods

All chemicals, reagents, and solvents were purchased from Sigma–Aldrich (Saint Louis, MO, USA) and used as received

### 2.1. Synthesis

The α-aminophosphonate compounds were prepared according to the one-pot procedure described previously by the authors via the Kabachnik-Fields reaction [24]. The corresponding amine (1 eq.) was then dissolved in solvate ionic liquid (SIL) [Li(G3)]TFSI (0.5 mL) with gentle heating. To the resulting mixture, the corresponding benzaldehyde (1 eq.) was added and stirred at room temperature for 5 min before the addition of diphenyl phosphite (1.2 eq.), and then, the mixture was stirred for an additional 25 min. At the conclusion of this time, the mixture was dissolved in diethyl ether (20 mL) and added to water. The diethyl ether was removed under reduced pressure to afford a suspension of precipitate in the aqueous phase, which was then filtered and washed with excess water and petroleum spirits (40–60 °C). Dissolution in diethyl ether, addition to water, and removal of organic solvent were repeated to analytical purity. The solid compound was collected and analysed by ^1^H NMR.

### 2.2. Sample Preparation

The individual compounds were introduced to the epoxy/amine system as a weight percentage (20%), in place of the DDM hardener (e.g., the control required 1.2 g of DDM, and in the modified samples, 0.96 g of DDM and 0.24 g of compounds 5–10 was used. Therefore, each compound was used in substitution of DDM by 20 wt %.). The compounds were each weighed out and heated until melted, and then, they were stirred with melted DDM until dissolved, prior to the addition to warm DGEBA. The mixture was thoroughly stirred until homogenous, and then, it was poured into required moulds. The curing cycle involved 2 h at 100 °C, 2 h at 120 °C, followed by post cure of 4 h at 175 °C, consistent with the prescribed process for this resin system. Table 1 outlines the mass of resin, hardener, and compound used in the samples and subsequent percentage of phosphorus content.

### 2.3. Analytical Techniques

#### 2.3.1. Nuclear Magnetic Resonance Spectroscopy (NMR)

All ^1^H, ^13^C, ^19^F, and ^31^P NMR spectra were recorded on a Bruker AVANCE III 500 MHz (Massachusetts, MA, USA) or Bruker AVANCE 400 MHz (Massachusetts, MA, USA) as indicated. Samples were dissolved in deuterated DMSO or deuterated chloroform (CDCl_3_) with the residual solvent peaks used as an internal reference (DMSO-d_6_: δ 2.50 ppm and CDCl_3_: δ 7.26 ppm).

#### 2.3.2. Infrared Spectroscopy

Near infrared spectroscopy (NIR) was performed on a Bruker Alpha FT-IR (Massachusetts, MA, USA) in transmission mode at a resolution of 4 cm^−1^ and an average of 128 scans between 4000 and 7500 cm^−1^.

#### 2.3.3. Thermogravimetric Analysis (TGA)

TGA was carried out in both oxidative and non-oxidative environments using a TA Instruments TGA Q50 analyser (New Castle, DE, USA). For air atmospheres, a flow rate of 60.0 mL min^−1^ was used, and for those performed in nitrogen, a flow rate of 40.0 mL min^−1^ was employed. Resin samples of 5–10 mg were heated from 20 to 800 °C in air and to 800 °C in nitrogen at a constant heating rate of 20 °C min^−1^.

#### 2.3.4. Limiting Oxygen Index

Ignition was performed using a lighter flame applied to the end face of the sample. The flame was applied for 10 s in order to induce uniform burning across the top. Samples were considered ignited when the flame lasted for more than 30 s. Pure oxygen (99.9%, O_2_) and nitrogen (99.99%, N_2_) streams in combination with mass-flow controllers (Bronkhorst F-203AV, Ruurlo, Netherlands) were used to achieve target oxygen concentrations. All tests were conducted with a nominal flow velocity of 100 mm s^−1^. The test condition temperature was 23 ± 2 °C. Reported LOI values are defined as the lowest oxygen concentration in which a given sample did not extinguish for 180 s from the moment of ignition. The ASTM D2863 standard [25] suggests that the flaming should last > 180 s to calculate the LOI value.

#### 2.3.5. SEM

SEM was used to obtained images of the samples after LOI testing to investigate the morphologies post-burn. SEM imaging was performed on a Zeiss Supra 55-VP (Oberkochen, Germany) at an electron accelerating voltage (electron high tension) of 3 kV. The burnt samples were mounted to an aluminium pin stub using double-sided carbon tape. Imaging was performed with a 2 nm carbon coating to enhance the visualization.

## 3. Results and Discussion

### 3.1. Synthesis and Characterisation

**Figure 2:**
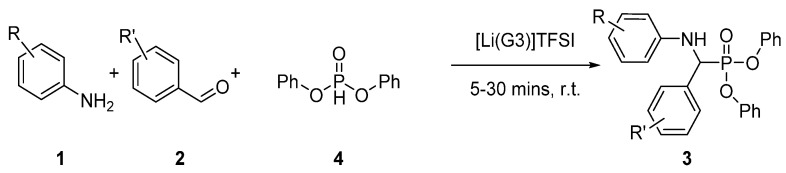
Synthesis of α-aminophosphonate analogues using Kabachnik-Fields in solvate ionic liquids.

A catalogue of seven α-aminophosphonate compounds were synthesized for investigation (Figure 2). The one-pot synthesis involved the in situ formation of an imine from the corresponding amine-benzaldehyde, before introducing the diphenyl phosphite.

**Figure 3:**
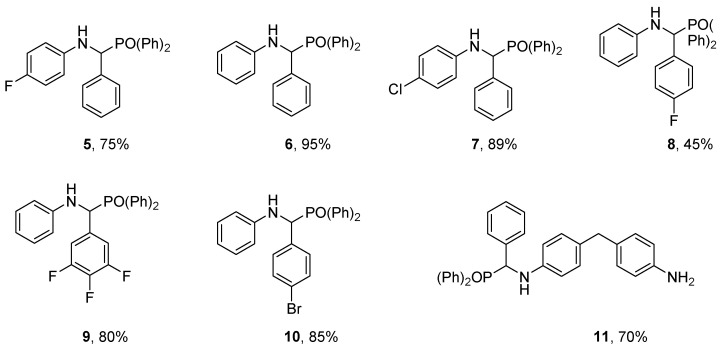
Compounds synthesised and corresponding yields for novel compounds.

The reaction proceeds to completion in a maximum of 30 min at room temperature, yielding the corresponding α-aminophosphonate compounds (Figure 3). Mono-substitution of the parent diamine was achieved giving 11, to compare the ability of the unreacted primary amine and secondary amine to react with the epoxy matrix and facilitate covalent integration into the network. The single substitution was prepared through careful stoichiometric regulation.

The efficiency of each halogen atom (bromine, chlorine, and fluorine) is compared to assess the abilities to act synergistically with phosphorus to impart flame retardant properties to the polymer system. Compounds 6 and 11 are without halogen substitution to compare the abilities of phosphorus alone.

**Figure 4:**
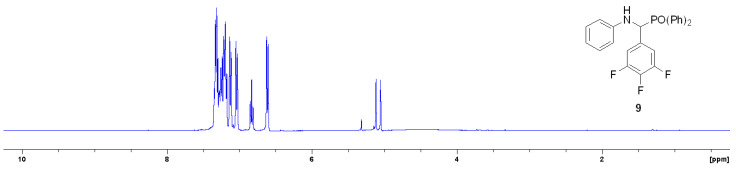
^1^H NMR spectra of 9 in CDCl_3_.

Each analogue was analysed by ^1^H NMR. The spectra of new derivative 9 is provided below (Figure 4). The doublet in the ^1^H NMR spectrum at δ 5.15 ppm is diagnostic of the α-aminophosphonate as it possesses the correct chemical shift and coupling constant (27 Hz) consistent with a J_2_ hydrogen–phosphorus interaction.

#### Chemical Structure of the Cured Network

**Figure 5:**
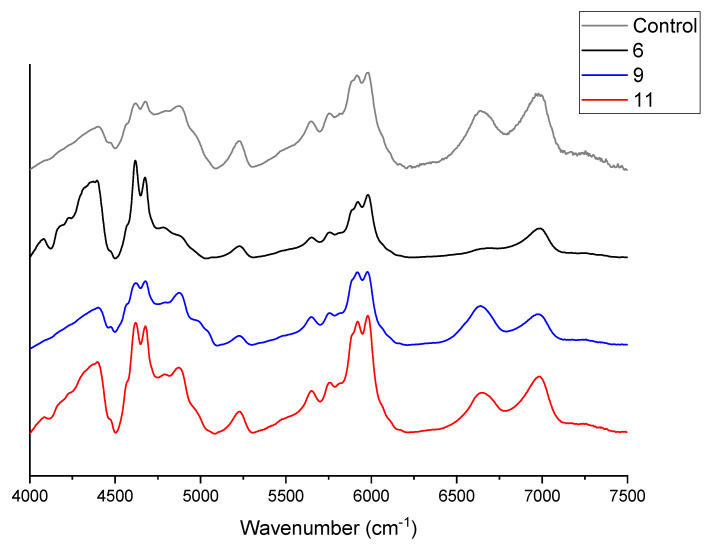
NIR analysis of DGEBA/DDM resin doped with samples (grey is the control spectra). NIR spectra of each substitution to evaluate amine incorporation (small molecule α-aminophosphonate and DDM-substituted α-aminophosphonate). Blue bands indicating the presence of epoxy, amine, and hydroxyl peaks, respectively.

NIR was used to examine the cure mechanism and incorporation of the α-aminophosphonate compounds into the polymer network, by assessing the presence of an epoxide peak (~4530 cm^−1^) and the increase in hydroxyl absorbance (~7000 cm^−1^). Observation of a combination of primary and secondary amine absorbance (~6570–6670 cm^−1^) identifies presence of these species in the system (Figure 5).

Figure 5 compares the NIR analysis of the α-aminophosphonate analogues to observe if the degree of curing has been disturbed due to the addition of the compounds. The grey curve corresponds to the control sample, and minimal epoxide peaks are present (~4530 cm^−1^) with high hydroxyl absorbance (~7000 cm^−1^), indicating that there is negligible unreacted epoxy in the system. A primary/secondary amine peak observed at ~6640 cm^−1^ is expected, as the addition ratio of epoxy/amine introduces an excess of amine. Examination of the NIR spectra for each of the samples showed trace amounts of residual epoxide (observed at ~4490 cm^−1^); the large presence of hydroxyl absorbance (observed at ~7000 cm^−1^) also indicates that the epoxy in the network has undergone cross-linking. The sample containing compound 11 (red spectra) shows a presence of a combination of primary and secondary amines; this is expected as there is an excess of amine in this system compared to the other α-aminophosphonate compounds. The sample containing compound 6 (black spectra), without any halogenated substitution, does not reveal the presence of residual epoxide or amine, indicating that the sample is cross-linked without unreacted primary or secondary amine.

### 3.2. Thermogravimetric Analysis and Flammability

The cured resin samples were tested via TGA to assess their thermal stability, in both air and nitrogen atmospheres. The resulting TGA curves (in air) are demonstrated below, identifying which substituents from each reagent component (i.e., originating from the aniline or benzaldehyde) are optimal for imparting fire retardance.

**Figure 6:**
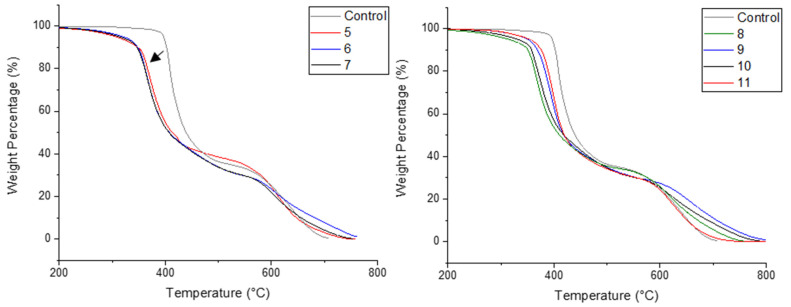
TGA curves in air, arrow indicating an earlier onset of degradation.

The TGA curves of all derivatives in air (Figure 6) demonstrate an earlier onset of degradation in comparison to the control sample, suggesting promotion of char formation and a wider plateau range, highlighting an increased stability at high temperatures.

α-aminophosphonate 5 bearing a 4-fluoro substituent showed that the Compounds 9 and 10 possess a superior elongating tail, demonstrating an improved resistance to thermal degradation.

All samples have an extended plateau ranging between 420 and 620 °C, which is an improvement on the plateau range of the control samples (460–590 °C).

**Figure 7:**
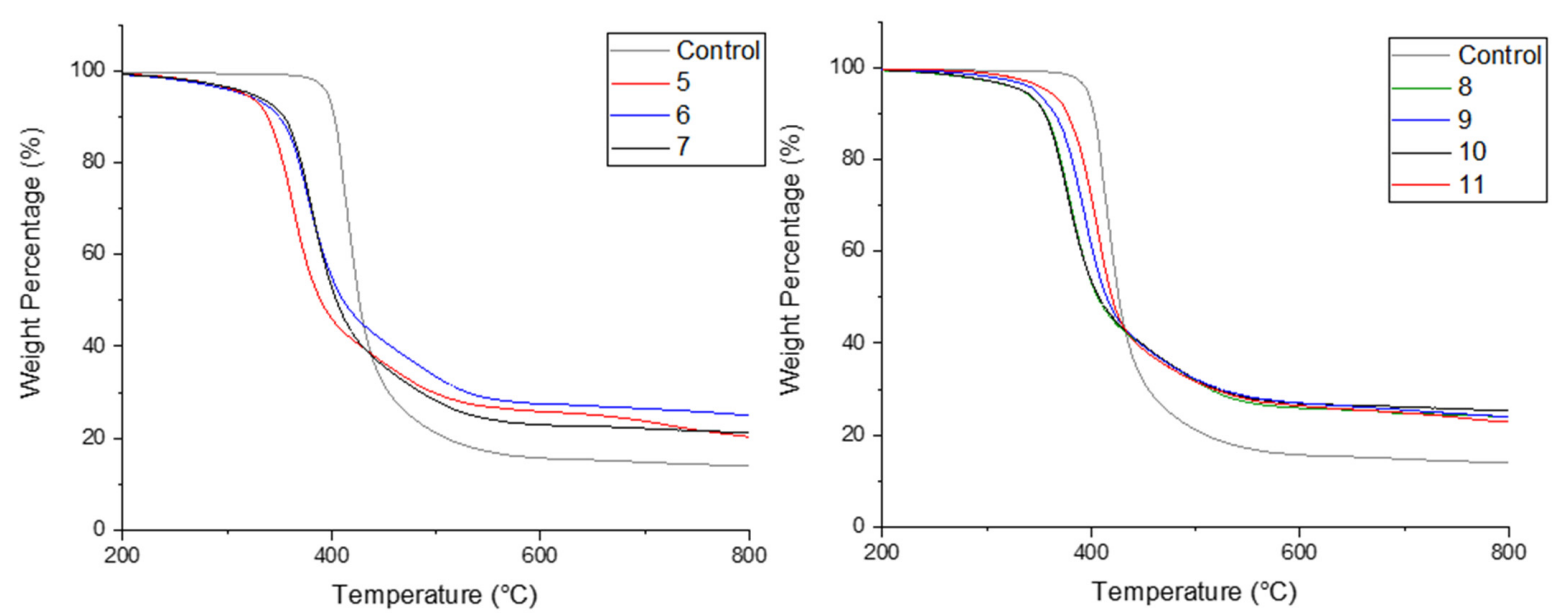
TGA curves in inert atmosphere.

Figure 7 demonstrates the TGA curves in inert atmosphere. Improvements in the inert atmosphere TGA for these compounds were also observed with higher residual weights at 600 °C for each compound. 

Of particular note is 5, which showed excellent improvements in both nitrogen and air (Table 2). This is interesting as α-aminophosphonate 9, bearing the 3,4,5-fluorine substitution, did not show a similar behaviour. This would suggest that the introduction of more fluorine atoms does not further improve the fire-retardant properties of the polymer material and perhaps a balance between halogen content and beneficial flame retardant properties exists in this scaffold.

To assess the minimum oxygen concentration to sustain flaming combustion, of each sample with the incorporation of the α-aminophosphonates, the LOI was determined (Table 2). This test involves defining the minimum concentration of oxygen (expressed as a percentage) at which a material can sustain a flame. The LOI for the control resin was determined to be 28.0% ± 0.3%. The addition of 8 resulted in the largest increase to 34.6% ± 0.3%, followed by 5 that achieved an LOI of 34.2% ± 0.2%, demonstrating that the introduction of *para*-fluorine substitution provides the highest improvement.

Interestingly, compound 9, the trifluoromethyl derivative, does not provide further improvement, with the lowest LOI increase (30.0% ± 0.1% relative to the remaining analogues), consistent with thermogravimetric analysis. This compound, (9), also demonstrated high amounts of unreacted amine and residual epoxy present in the sample (Figure 5). This could be due to the 4-fluroine deactivating the scaffold, thus minimizing its inclusion into the polymer network.

Moreover, the compound bearing the 3,4,5-fluorine substitution is less soluble, inducing phase separation and impacting the ability of the compound to homogenously incorporate into the polymer material.

The introduction of bromine or chloride did not prove substantial benefit over the compounds without any substitution (33.0% ± 0.4%, 32.2% ± 0.2%, and 32.8% ± 0.3%, respectively).

The maximum improvement (relative to control sample) obtained was 6.6%. This is a considerable increase and furthers our previous work, which obtained overall improvement of 2.3% [23]. Moreover, the LOI values reported herein are higher when comparing to studies that also modified DGEBA/DDM resin systems [9,10,26].

### 3.3. Morphology of the Residue

**Figure 8:**
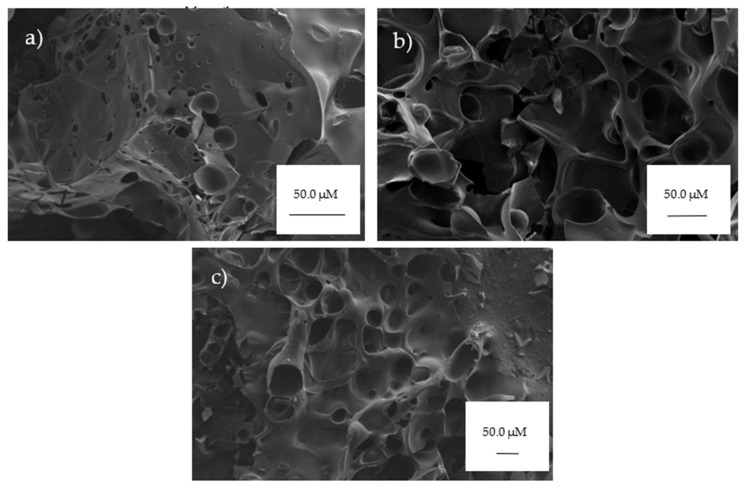
SEM images after LOI testing: (**a**) control, (**b**) sample containing compound **8**, and (**c**) sample containing compound 11.

After LOI testing, the morphology of the residue was investigated by SEM. Figure 8 displays the morphologies of the residues from the control sample (DGEBA/DDM) and the resin samples containing compound 8 and 11. The morphology of the control resin displays a surface with some small holes and smooth patches, due to the complete burning. In comparison, the morphologies of the charred resin samples containing compounds 8 and 11 exhibit porous interior morphology with pores and bubbles. This indicates that the incorporation of the α-aminophosphonate compounds restricts the rapid volatilization at the surface and they act as an insulating layer

In conclusion, we have reported the rapid, easily accessible synthesis of a catalogue of α-aminophosphonates to assist in the covalent integration to an epoxy-based resin system. NIR confirmed the incorporation of the compounds to the polymer system, demonstrating minimal unreacted epoxy with strong hydroxyl contribution. The compounds assisted in imparting flame retardance, with improved thermal degradation as verified by TGA and LOI. This is further exhibited in SEM images of the burnt residue, revealing the protective effect of the char layer to the underlying polymer. The compounds bearing para-fluorine substitution provided the highest improvement, with no statistical difference between bromine and chlorine substitutions to the control. This demonstrates the synergistic effects of incorporating phosphorous- and fluorine-containing compounds into epoxy resin systems to decrease flammability.

## Figures and Tables

**Table 1:** Amounts of hardener, resin, and compound with phosphorus content.

Compound	Resin (g)	Hardener (g)	Compound (g)	P %
Control	1.8	1.2	0	0
5–10	1.8	0.96	0.24	~0.60
11	1.8	0.96	0.24	0.48

**Table 2:** TGA char yield and LOI data.

Compound	Char Yield (%) ^a^	Char Yield (%) ^b^	LOI (%)
Control	35.8	21.1	28.0 ± 0.3
5	38.5	29.6	34.2 ± 0.2
6	33.8	33.2	32.8 ± 0.3
7	33.7	28.1	32.2 ± 0.2
8	35.4	31.6	34.6 ± 0.3
9	33.5	30.8	30.0 ± 0.1
10	33.1	32.0	33.0 ± 0.4
11	36.0	31.0	33.2 ± 0.5

^a^ Analysis conducted in air, ^b^ analysis conducted in nitrogen, char yield at 500 °C.

## Data Availability

Data sharing is not applicable.
